# Construction of an Efficient Engineered Strain for Chaetoglobosin A Bioresource Production from Potato Starch Industrial Waste

**DOI:** 10.3390/foods14050842

**Published:** 2025-02-28

**Authors:** Kai Zhang, Shanshan Zhao, Zhengran Wang, Ming Cheng, Wan Wang, Qian Yang

**Affiliations:** 1 Donghai Laboratory, Zhoushan 316021, China; zhangkaiown@163.com; 2 School of Life Science, Ludong University, 186 Hongqi Road, Yantai 264025, China; 3 School of Life Science and Technology, Harbin Institute of Technology, Harbin 150080, China; glxmdj@163.com (M.C.); 17862702273@163.com (W.W.); 4 Institute of Marine Biology and Pharmacology, Ocean College, Zhejiang University, Zhoushan 316021, China; wangzhengran202106@163.com

**Keywords:** *Chaetomium globosum*, bioresource production, engineered strain, CheA

## Abstract

Chaetoglobosin A (CheA), a typical structure of the cytochalasin family, exhibits outstanding efficacy against a variety of tumor cells and plant pathogens. However, its low yield and high production cost are major obstacles limiting its wide application. In order to increase CheA yield, an engineered strain was established by overexpressing *CgMfs*, the gene encoding the MFS family’s efflux pump, on chassis cells lacking *CgXpp1*, which have been shown to act as a negative regulator of CheA biosynthesis. As expected, the engineered strain significantly boosted CheA production from 63.19 to 265.93 mg/L after incubation in PDA medium for 10 d, whereas the yield of the engineered strain was remarkably enhanced 2.93-fold compared with the wild type, following 10 d of cultivation utilizing potato starch industrial waste. The addition of metal ions had a positive effect on CheA production, with Cu^2+^ being the most effective and improving production to 176.92 mg/L. The optimal fermentation conditions were determined by response surface optimization, and under the optimal conditions, the engineered strain could stably produce CheA with a yield of 197.58 mg/L. This study provided the conditions for reducing production costs while increasing CheA production, as well as new strategies and insights for the production of the target compound.

## 1. Introduction

According to the Food and Agriculture Organization of the United Nations (FAO), China is the largest potato producer with 95.63 million tons produced annually [1]. A recent market study reported that the global potato starch market reached 3.9 million tons in 2020 and 4.4 million tons in 2024 [2]. In addition, the global potato starch market is expected to grow from USD 3.49 billion in 2023 to about USD 5 billion in 2030 [3]. Although the potato starch industry brings high economic returns, it also brings environmental pollution problems.

The global potato starch industry produced about 10.8 million m^3^ of wastewater in 2017; this will increase to 12 million m^3^ by 2023, constituting about one-third of wastewater [4]. The industrial by-products of potato starch contain a lot of pectin, fiber, protein, and various bioactive substances, showing an industry with large by-product production, low resource utilization, and serious environmental pollution [5]. The wastewater produced during potato starch production has a pH range of 3.9–7.5, is rich in organic compounds (e.g., 19–25 g/L starch, 2.8–4 g/L protein, and 0.3 g/L glucose) and inorganic salts (e.g., containing 0.1–751 mg/L nitrogen, 128–361 mg/L phosphorus, and 1613–2222 mg/L potassium), and has a chemical oxygen demand of 6000–30,000 mg/L [6,7,8,9]. Direct discharge into the environment has the risk of the eutrophication of water bodies due to the overgrowth of phytoplankton and aquatic plants [10]. Therefore, the discharge of this waste, which has proven harmful to ecosystems, must be purified to meet nationally recognized standards.

In recent years, there has been increasing interest in biotechnological approaches using industrial by-products as components of microbial media. These methods can achieve the complete biodegradation of organic compounds and produce new products with added value [2,11]. In addition, using industrial by-products as a medium for microorganisms can effectively reduce total production costs. Therefore, the recovery and utilization of potato starch industrial by-products through biotechnology can produce products with biological activity and application value, which not only increases economic benefits but also reduces the environmental pollution from a large number of by-products.

As an important biocontrol strain, *Chaetomium globosum* is well known for its broad-spectrum antibacterial activity against a variety of plant pathogenic fungal strains; for example, it has good inhibitory effects on *Fusarium porotrichioides*, *Setosphaeria turcica*, and *Rhizopus stolonifer*. It has also received extensive attention among numerous researchers due to its capacity to generate a diverse range of bioactive constituents, including extracellular hydrolases, polysaccharides, and active secondary metabolites [12,13,14,15,16]. Notable among these is chaetoglobosin A (CheA) of the cytochalasin family, which is generated through the catalysis of polyketide synthase (PKS). It not only demonstrates highly efficient antibacterial activities against plant-parasitic nematodes and pathogenic fungi, but also exhibits broad-spectrum cytotoxicity toward inflammatory cells and tumor cells [17]. First reported in 1973, CheA is the most abundant member of the cytochalasin family and has attracted significant attention due to its diverse biological functions [17,18]. As a crucial substitute for chemical reagents, research on chaetoglobosin bioactive substances holds great significance for agricultural production. It has been 40 years since Sekita discovered CheA, and our team is committed to the study of CheA biosynthesis. We have verified the influence of several key synthetic genes and regulatory genes of CheA on its yield, but the high production cost is still the main factor limiting its widespread use [12,18,19,20,21,22].

There have been few studies on the production of CheA from potato starch industrial waste. Previous studies found that *C. globosum* effectively degrades refractory compounds such as lignin, indicating that it is possible to use cellulose and other components in potato starch industrial waste [15,20,23]. Therefore, using potato starch industrial waste as the only fermentation substrate, the *CgXpp1* mutant strain, with the knockout of the *CgXpp1* feedback regulator, was used as chassis cells to overexpress the CgMfs gene encoding CheA transport and to construct an engineered strain with a high yield of CheA. On this basis, to further improve the production performance of the engineered strain and enhance the yield of the active metabolite CheA, the fermentation conditions of the engineered strain will be optimized using the response surface methodology. The attempt to establish a low-cost and high-efficiency production scheme for CheA can greatly promote the industrial production process of the compound and provide a more theoretical basis for this. This is an environmentally friendly solution for the use of potato starch industrial waste and a sustainable source of raw material for CheA production.

## 2. Materials and Methods

Restriction enzymes were purchased from Thermo-Fisher (Waltham, MA, USA). TaqDNA polymerase and the HiScript II 1st Strand cDNA Synthesis Kit were purchased from Vazyme Biotech Co., Ltd. (Nanjing, China). *C. globosum* was preserved in our laboratory, and *Escherichia coli* DH5α was purchased from Beijing Tsingke Biotech Co., Ltd. (Beijing, China). Lywallzyme was acquired from the Institute of Microbiology, Guangdong Academy of Sciences (Guangzhou, China). All chemicals and organic reagents utilized in the experiments were of analytical grade.

### 2.1. Construction of Engineered Strains

#### 2.1.1. Construction of Mfs Overexpression Vector

Chassis cells with a dysfunctional *CgXpp1* gene were constructed by Zhao [18]. The plasmid pBARGPE1-mCherry, which contains a bialaphos resistance gene and a red fluorescent screening marker, was selected as the overexpression skeleton. The complete *CgMfs* gene fragment was amplified by PCR using oligonucleotide primers mcheOF-CgMfs/mcheOF-CgMfs (Table S1). The PCR reaction system was 20 μL: 1 μL for both primers and template, 2× mix was used (10 μL), and they were supplemented to yield 20 μL with ddH_2_O. Subsequently, the linearized vector (digested with *BamH*I and *EcoR*I) and the *CgMfs* fragment, which included 20–25 bp homologous regions on both sides, were ligated following the instructions provided with the ClonExpress MultiS One Step Cloning Kit (Vazyme, Nanjing, China). The gene engineering transformation vector was then obtained, verified, and utilized for subsequent transformation experiments.

#### 2.1.2. Transformation and Screening of Mutant Strains

According to Zhang’s methodology, *Chaetomium globosum* was inoculated into a potato dextrose agar (PDA) liquid medium and subjected to incubation under constant temperature oscillation at 180 rpm for two days [18]. The mycelia were subsequently harvested and rinsed with sterile water. To minimize residual moisture, which could potentially dilute the effective concentration of Lywallzyme, the mycelia were blotted with sterile filter paper before being transferred to a sterile 50 mL Erlenmeyer flask. A solution of 0.15 g Lywallzyme was prepared by dissolving it in 10 mL of sterile 0.7 M NaCl solution within a 15 mL centrifuge tube. This solution was then filtered through a 0.22 µm membrane and added to the flask containing the mycelia. The mixture was incubated at 30 °C with agitation at 110 rpm to facilitate digestion. The release of protoplasts was monitored and documented at 30 min intervals, with the digestion process typically lasting between 2.5 and 3 h. Subsequent experiments were initiated once the protoplast concentration reached 1 × 10^6^. The resulting protoplasts were separated by filtration through a single layer of sterile filter paper, and the precipitate was collected via centrifugation. The protoplasts were then thoroughly rinsed with 0.7 M NaCl solution and STC buffer, respectively. The morphology of the protoplasts was examined using microscopy, and their concentration was quantified with a hemocytometer. The protoplasts were suitably diluted to optimize the transformation efficiency. Subsequently, the protoplasts were combined with a constructed plasmid vector (5–10 µg) and incubated in an ice bath for 20 min. Polyethylene glycol 4000 (PEG4000, Thermo Scientific, Waltham, MA, USA) facilitated the protoplast transformation, with sequential additions of 200 μL, 200 μL, and 800 μL at 30 min intervals. The transformation mixture was then combined with freshly prepared sterile regeneration medium and distributed onto plates for overnight incubation at 28 °C. The following day, a resistance screening layer consisting of potato dextrose agar (PDA) with 1% agar and an appropriate antibiotic concentration was applied. A control plate, lacking the resistance screening layer, was utilized to assess the regeneration efficiency of the protoplasts.

### 2.2. Determination of Potato Waste Components

For the analysis of potato waste, samples were dried in an oven at 70 °C for 24 h followed by 110 °C for 2 h. Their mass was measured, and their dry matter content was calculated. Starch content was determined using the enzymatic hydrolysis method. The sample underwent pulverization, followed by the addition of amyloglucosidase to facilitate hydrolysis. The starch content in the resulting solution was subsequently estimated through the calibration of an alkaline tartrate copper solution. The Van Soest method was employed to extract and quantify the cellulose, hemicellulose, and ash content [24]. Total protein and fat contents were determined using the Kjeldahl method and Soxhlet extraction, respectively.

### 2.3. Determination of CheA

The CheA standard product, procured from Sigma-Aldrich (Darmstadt, Germany), was dissolved in methanol and prepared into standard reagents of varying concentrations using liquid chromatography with a C18 reverse-phase column (Agilent (Santa Clara, CA, USA), 4.6 mm × 250 mm, 5 μm) from Waters Company (Milford, MA, USA). The mobile phase consisted of an acetonitrile solution containing 55% pure water. The chromatographic conditions included a flow rate of 1.0 mL/min and a detection wavelength of 227 nm, with three parallel standard curves constructed for each group. The fermentation sample was subjected to centrifugation, after which the supernatant was extracted with an equal volume of ethyl acetate. The ethyl acetate layer was collected, dehydrated using anhydrous sodium sulfate, and concentrated via rotary evaporation. Following its concentration, the sample was dissolved in 1 mL of methanol. Subsequently, it was filtered through a 0.22 µm organic filter membrane, and the concentration of CheA was determined.

### 2.4. Quantitative Analysis of Transcription Levels

Utilizing Wang’s methodology, a quantitative PCR kit from Promega (Beijing, China), featuring SYBR Green as a fluorescent marker and the *β*-actin gene as an internal reference, was employed to quantitatively assess the relative expression levels of the target genes at various time points across different mutant strains. The expression levels were analyzed using the 2^−ΔΔCT^ method [19]. Each group included three replicates to ensure data accuracy.

### 2.5. Effects of Metal Ions on the Yield of CheA

To enhance the production of CheA from potato waste by *C. globosum*, the influence of metal ions K^+^, Na^+^, Mg^2+^, Fe^2+^, Zn^2+^, Cu^2+^, and Ca^2+^ on CheA yield was investigated. A concentration of 200 mg/L of metal ions was added to 5 g/L of potato waste, and CheA yield was measured as described in Section 2.4.

### 2.6. Response Surface Optimization

To enhance the yield of CheA, we systematically optimized the fermentation conditions for the engineered strain to produce CheA, utilizing potato waste as a substrate. The parameters investigated in the one-factor experiment included fermentation pH levels (5, 6, 7, 8, and 9), fermentation temperatures (24, 26, 28, 30, and 32 °C), rotational speeds (120, 150, 180, 210, and 240 rpm), dry matter content of potato waste (2.5, 5.0, 7.5, 10.0, and 12.5 g/L), and inoculation amounts (0.5, 1.0, 1.5, 2.0, and 2.5%). Subsequently, response surface optimization was conducted focusing on fermentation temperatures (26, 28, and 30 °C), fermentation pH levels (5.0, 6.0, and 7.0), rotational speeds (160, 180, and 200 rpm), and dry matter content of potato waste (2.5, 5.0, and 7.5 g/L). Design-Expert 8.0 software was used to fit the multiple regression of the experimental data, resulting in a binary multiple regression model for the genetically engineered strains concerning the aforementioned factors. The optimal fermentation conditions were determined through the analysis of response surfaces and contour plots.

### 2.7. Amplification Fermentation

The fermentation parameters for the expanded culture were specified as follows: a temperature of 28.0 °C, a pH level of 6.0, a ventilation rate of 0.20 vvm, an addition of 5.0% potato waste, an inoculation volume of 1.0%, and a liquid loading volume of 5.0 L.

### 2.8. Data Analysis

All data were derived from a minimum of three replicates, and results were expressed as the mean and standard deviation of independent experiments. Statistical analyses were conducted using Student’s *t*-test and one-way ANOVA for independent sample comparisons. A *p* < 0.05 was considered statistically significant, denoted by *, while a *p*< 0.01 indicated a highly significant difference, denoted by **.

## 3. Results and Discussion

### 3.1. Construction of Engineered Strain

**Figure 1:**
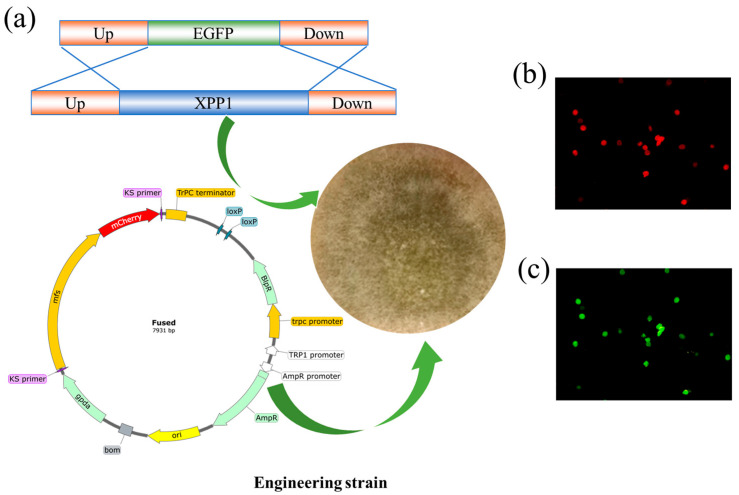
The construction of engineering strain and its verification. (**a**) The construction of engineering strain. (**b**,**c**) Phenotypic characterization of transformants.

Previous studies have found that *Xpp1* transcription negatively regulates CheA biosynthesis and demonstrated that MFS transfers CheA from intracellular to extracellular sides [18,19]. Therefore, we used a strain with a knocked-out *CgXpp1* feedback regulator as a chassis cell to overexpress the *CgMfs1* gene, encoding the MFS family transporters, to generate a high-yielding genetically engineered strain of *C. globosum* (Figure 1a). The *pmCherry–CgMfs* was transformed into the *Xpp1* deletion strain by PEG4000-mediated protoplast transformation, and the positive clones were screened by employing their resistance to bialaphos and the red fluorescent protein gene in the *pmCherry–CgMfs* vector. After culturing, the transformants were prepared with a spore suspension, part of which was used for strain preservation (−80 °C) to prevent genetic instability caused by multiple generations. The other part was used for the multilevel verification of transformants. The mutant strains were inoculated in PDA and cultured at 28 °C for 9 d. The genome was extracted and the primers HPF/HPR were used to determine the DNA levels of the transformants. Furthermore, the *Xpp1* knockout strain exhibited green fluorescence using an inverted microscope due to the presence of the *EGFP* gene (Figure 1b), while the overexpression of *Mfs* results in red fluorescence, attributed to the inclusion of the *Cherry* gene in the vector (Figure 1c).

### 3.2. CheA Production in the Engineered Strain

**Figure 2:**
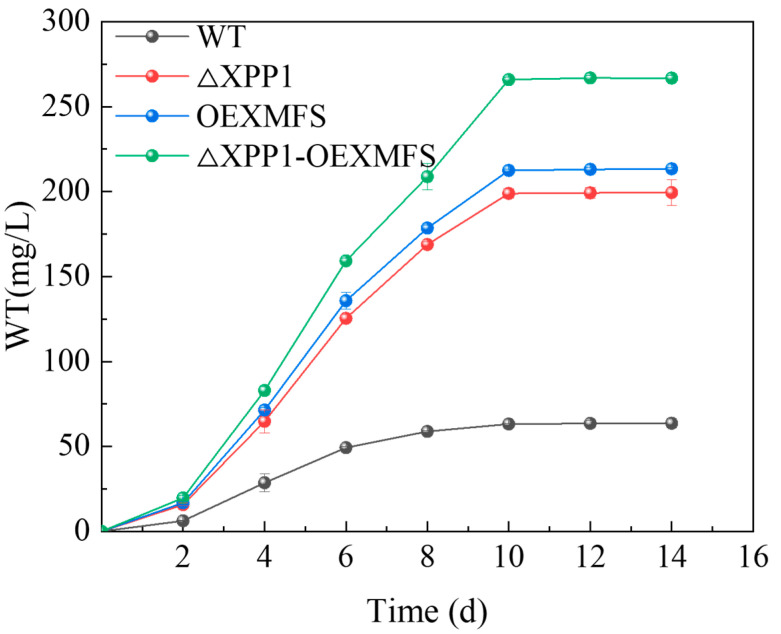
Effect of different times on the yield of CheA.

To investigate the effect of engineered *C. globosum* with *Xpp1* knockout and *Mfs* gene overexpression on the CheA yield, the CheA yield of each strain is shown in Figure 2. With the extension of the fermentation time, within the range of 0–10 d, the amount of CheA biosynthesis by each strain also showed an increasing trend. The WT reached 63.19 mg/L at 10 d, and the *Xpp1* knockout and *Mfs* overexpression strains were 198.93 and 212.45 mg/L, respectively, indicating that both *Xpp1* knockout and *Mfs* overexpression could increase CheA yield. The CheA yield for the engineered strain was 265.93 mg/L after 10 d of incubation, which was significantly higher than that of the WT and other mutant strains, indicating that the overexpression of *Mfs* with *Xpp1* knockout had a synergistic effect on the CheA yield, but it was not a simple superposition effect. As the fermentation time continued to increase to 14 d, the CheA yield of each strain remained unchanged or showed a declining trend. Because the nutrients in the medium were consumed, the growth of the strain was inhibited or it even died, so the CheA yield tended to be balanced.

### 3.3. Transcription Levels of Genes Related to CheA Synthesis

**Figure 3:**
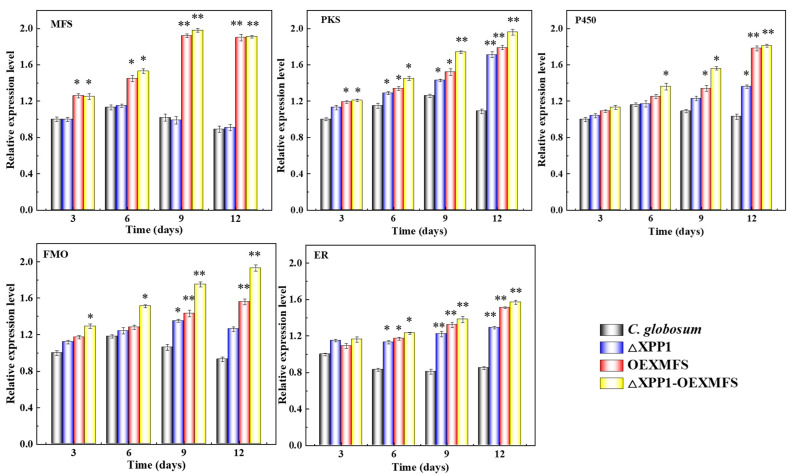
The effect of different times on the expression levels of the key synthesis gene (MFS, PKS, P450, FMO and ER) of CheA in mutant strains. (*: *p* < 0.05; **: *p* < 0.01).

To further investigate the effect of engineered strains on CheA biosynthesis, the expression levels of key synthetic genes (*CgP450*, *CgFMO*, *CgPKS*, and *CgER*) and the transporter gene (*CgMfs*) of CheA were each determined at 3, 6, 9, and 12 d. The expression levels of each gene during 3 d of WT fermentation culture were assigned as 1.0, with *β-actin* used as the internal reference gene for quantitative analysis (Figure 3). The expression levels of *CgMfs* and *CgER* in the WT culture decreased with increased culture time, and the expression levels of key genes in the mutant and engineered strains increased with increased fermentation time, among which the *CgMfs* expression levels in the engineered strain increased the most (1.98 times that of the WT culture at 9 d). The expression levels were similar to those of the *CgMfs-*overexpressing strain. In addition, at 12 d, the expression levels of *CgPKS*, *CgP450*, *CgFMO*, and *CgER* were 1.96-, 1.81-, 1.93-, and 1.57-fold those of the WT culture, which was the main reason for the significant increase in CheA.

### 3.4. Components of Potato Starch Industrial Waste

To better use potato starch industrial waste, we determined the main nutrients in potato starch industrial waste residue and wastewater (Table 1). There was 27.25 g of crude fiber, 5.73 g of protein, and 26.8 g of carbohydrates per 100 g of waste dry matter; and every 100 g of wastewater contained 9.74 g of crude fiber, 17.2 g of protein, and 11.3 g of carbohydrates. In addition, the waste residue and wastewater were both rich in inorganic salt ions. These results indicated that the industrial waste of the potato starch was rich in nutrients such as carbon and nitrogen sources, which may cause environmental pollution if discharged untreated. If used as a substrate for producing high-value-added CheA, this could solve the problem of environmental pollution and reduce the cost of CheA.

### 3.5. Effects of Potato Starch Industrial Waste on C. globosum and CheA

**Figure 4:**
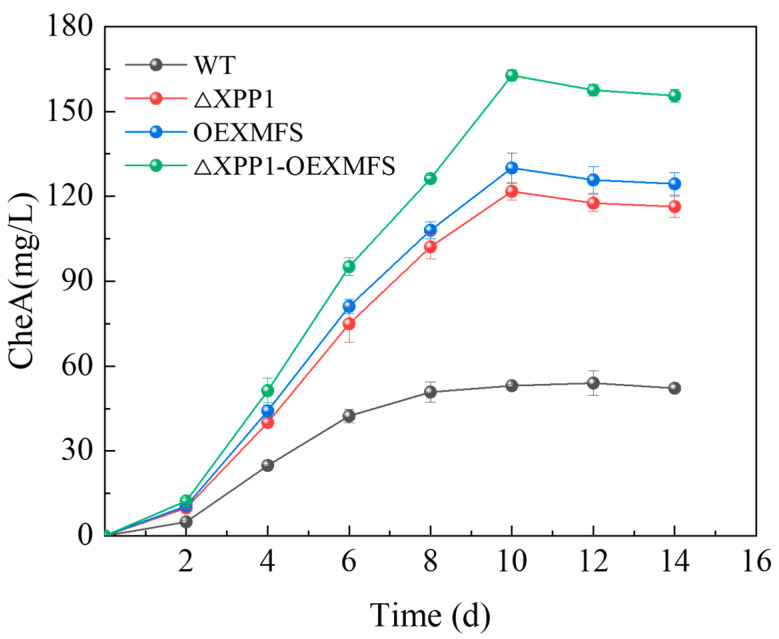
Production of CheA by each strain using the industrial waste of potato starch.

To further explore the use of potato starch industrial waste to produce CheA, we used potato starch industrial waste as the only substrate for fermentation experiments over different times (Figure 4). We found that *C. globosum* could make good use of the industrial waste of potato starch to produce CheA, and the trend of CheA production at different times was the same as in the PDA medium; however, the maximum value was significantly lower than in PDA. Among them, the WT strain reached 53.07 mg/L at 10 d; the *Xpp1* knockout and *Mfs* overexpressing strains were 116.33 and 124.43 mg/L, respectively, and the engineered strain reached 155.57 mg/L. Thus, the engineered strain with *Xpp1* knockout and *Mfs* overexpression could grow and produce CheA from potato starch industrial waste, but with some disadvantages compared with 265.93 mg/L in the PDA medium. In addition, we found that although the engineered strain could increase the production of CheA, it did not change its trend over time.

### 3.6. Effects of Different Metal Ions on C. globosum and CheA

**Figure 5:**
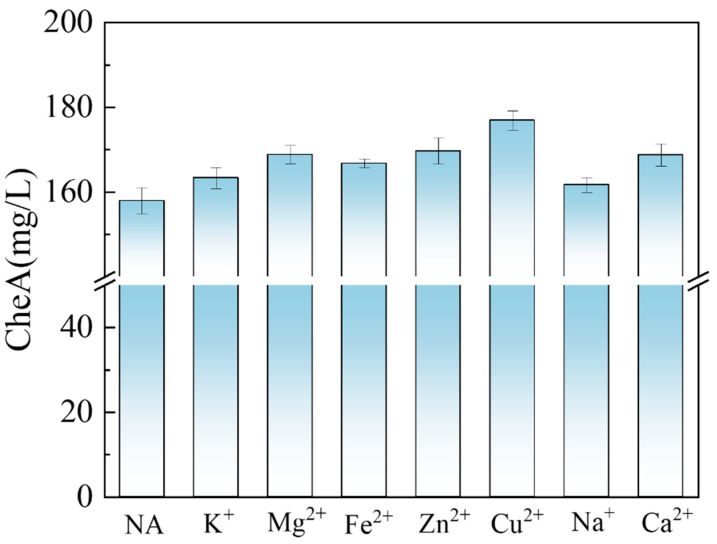
Effect of adding different metal ions on CheA yield.

Metal ions such as Zn^2+^, Cu^2+^, Fe^2+^, Fe^3+^, and Mg^2+^ play important roles in microbial metabolism, as cofactors or catalysts of enzymes, and participate in a variety of biochemical reactions [25,26,27,28]. Studies have shown that appropriate amounts of Zn^2+^ can increase the production of certain fungal secondary metabolites, but an excess may inhibit growth and metabolism. To improve CheA production from potato waste by engineered strains, we investigated the effects of adding different metal ions (K^+^, Na^+^, Mg^2+^, Fe^2+^, Zn^2+^, Cu^2+^, and Ca^2+^) in the medium on CheA production (Figure 5). Adding K^+^, Na^+^, and Fe^2+^ had no significant effect; a CheA yield of 157.93 mg/L was obtained without adding metal ions, indicating that these three ions had no promoting effect on the CheA yield. However, the addition of Cu^2+^, Zn^2+^, Mg^2+^, and Ca^2+^ significantly increased the CheA yield, among which Cu^2+^ increased the most, reaching 176.92 mg/L. It is believed that Cu ions can increase the permeability of the cell membrane and change the activities of PKS and CheA synthesis-related enzymes [29]. These findings highlight the importance of metal ions in microbial metabolism and their potential to optimize the production of valuable secondary metabolites such as CheA. Further research is warranted to elucidate the specific mechanisms by which these metal ions influence microbial enzyme activities and metabolite production, which could inform the design of more efficient bioprocessing strategies.

### 3.7. Effects of Fermentation Conditions on C. globosum Biomass and CheA

**Figure 6:**
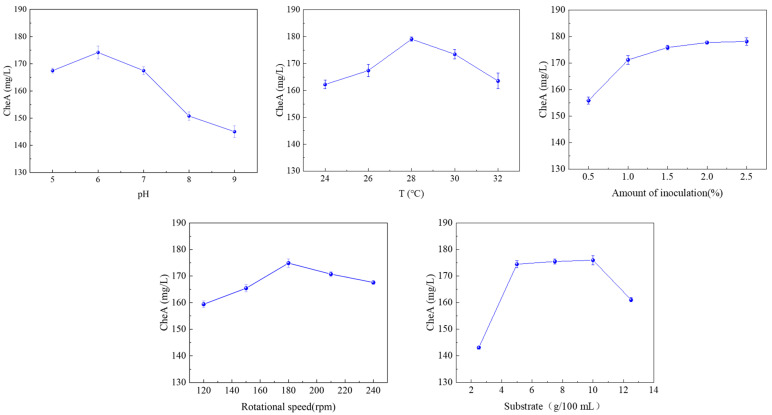
Optimization of fermentation conditions.

To further improve the CheA yield, we optimized the fermentation conditions for the engineered strain to produce CheA from potato waste (Figure 6). Environmental pH is very important for the growth and development of fungi and the synthesis of secondary metabolites [30,31]. Fungi exhibit pH-dependent metabolic regulation, with proton gradients and membrane potential directly affecting cellular processes such as nutrient uptake, enzyme catalysis, and metabolite export [32]. The CheA yield of the engineered strain also increased when the pH increased from five to six, with the highest yield of 174.17 mg/L for a pH of 6. However, when the pH was further increased beyond six, the CheA yield declined significantly, likely due to the disruption of cellular homeostasis, enzyme denaturation, and metabolic stress at higher pH levels.

Temperature is an important factor affecting *C. globosum* growth [33,34], and *C. globosum* is very sensitive to temperature, with the optimum fermentation temperature varying with the strain type, fermentation medium composition, and fermentation conditions. Generally, the optimum temperature for cell growth differed from that for optimum CheA production. With the increase in temperature, the CheA yield initially increased and then decreased, with the highest yield of 179.07 mg/L at 28 °C (Figure 6).

The amount of inoculation is also a key factor affecting the synthesis of natural products [33]. Increasing the amount of inoculation within a certain range can make the cell reach the equilibrium stage quickly and produce secondary metabolites [35]. To determine the best inoculation amount, the influence of an inoculation amount of 0.5–2.5% (*v*/*v*) on CheA production was investigated (Figure 6). The CheA yields initially increased and then decreased with the increase in inoculation rate. The 2% inoculation rate provided the highest CheA yield of 177.76 mg/L. The increase in CheA production was due to the increase in the initial microbial population in the fermentation system, which quickly passed the lag stage and increased CheA production. However, there was no increase in CheA production following further increases in inoculation, which may be because most of the nutrients in the medium are then used for *C. globosum* growth [36].

The rotational speed determines the oxygen supply and mixing levels of media components during fermentation, as continuous surface renewal helps maintain a concentration gradient between the inside and outside of the cell, ensuring the smooth and continuous transport of the matrix, other nutrients, and products across the cell wall [37,38,39]. However, too high a rotational speed produces high shear force, which may lead to cell damage, thus affecting cell growth and metabolite synthesis. The highest CheA yield of 174.88 mg/L was at 180 rpm (Figure 6).

The amount of substrate added was closely related to the growth and secondary metabolism of fungi [40]. We determined the influence of the substrate concentration (2.5–12.5 g/100 mL) on the CheA yield (Figure 6). The CheA yield increased from 143.05 to 176.02 mg/L as the substrate concentration increased from 2.5 to 5.0 g/100 mL. However, as the substrate concentration increased, the CheA yield initially remained unchanged and then declined. This trend suggests that while an adequate substrate supply is essential for metabolite production, excessive substrate concentrations may lead to catabolite repression, substrate inhibition, or nutrient imbalance, thereby reducing CheA synthesis. These findings highlight the importance of optimizing the substrate concentration to balance the engineered strain’s nutrient availability and metabolic flux [41,42].

### 3.8. Response Surface Optimization

To increase the CheA yield of the engineered strain using potato starch industrial by-products, the fermentation conditions were optimized, and 29 groups of experiments were designed according to the analysis using Design-Expert 8.0 software. The specific experimental schemes and results are shown in Table 2.

The regression and variance analysis (Table S2) showed an F-value of the model of 6144.16 (*p* < 0.001), indicating that the data fit well with the model and could be used to predict the experimental results. In addition, the F-value of the misfit was 0.21, and *p* = 0.9803 (i.e., >0.05), indicating that the error was not significant for this prediction model. In this model, A, B, C, D, AB, AC, AD, CD, A^2^, B^2^, C^2^, and D^2^ were important model terms, because all were *p* < 0.05. A multiple regression analysis of the experimental data resulted in the following second-order equation of CheA: CheA (mg/L) = 193.73 − 1.08 A − 1.27 B + 1.08 C − 1.17 D − 1.12 AB + 3.02 AC + 1.44 AD − 0.45 BC + 0.35 D − 0.75 CD − 23.15 A^2^ − 25.16 B^2^ − 24.76 C^2^ − 25.48 D^2^.

**Figure 7:**
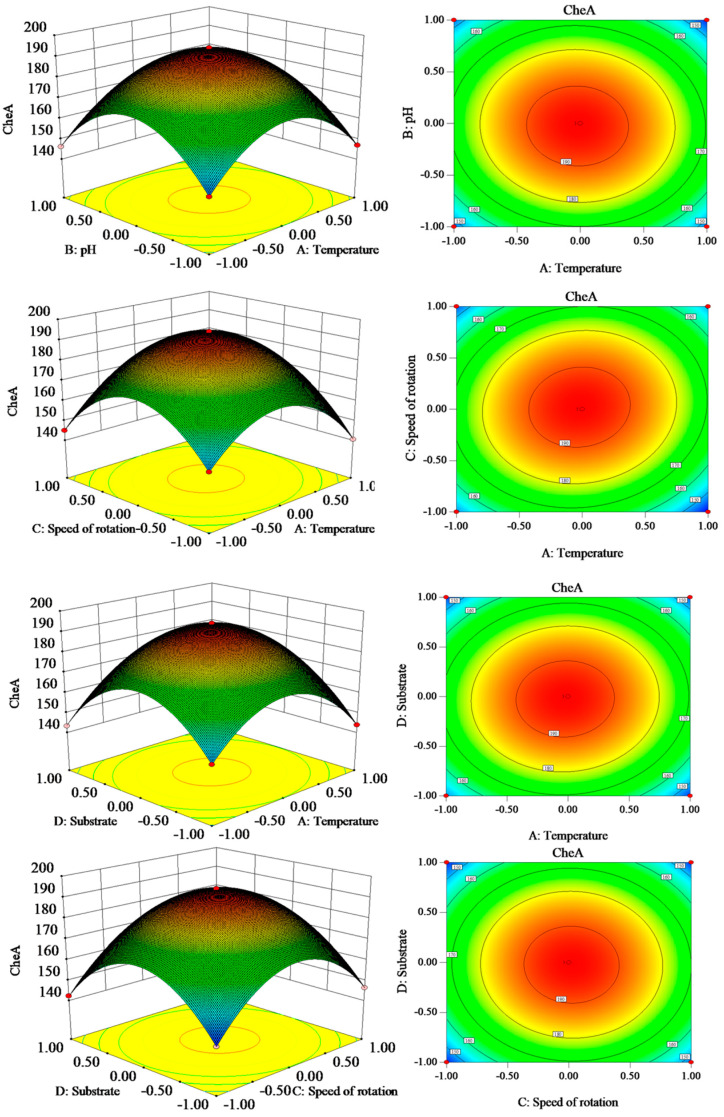
Contour map and 3D map of significant interactions.

The four factors also showed significant quadratic effects on CheA production at a significant (*p* < 0.05) temperature, which were pH, temperature–speed, temperature–substrate, and speed–substrate interactions. Figure 7 shows four contour maps and 3D maps of the significant interactions. The response surface provided the following optimum reaction conditions for the maximum CheA yield of 193.73 mg/L: a temperature of 28 °C, pH of 5.97, rotational speed of 180.02 rpm, and amount of added substrate of 4.98 g/L. Therefore, a temperature of 28 °C, pH of 6, rotational speed of 180 rpm, and substrate addition of 5 g/L were selected for three validation experiments. Under these conditions, the CheA yield was 193.67 mg/L, which was close to the predicted value, indicating accurate model prediction.

### 3.9. Scale-Up Experiment

**Figure 8:**
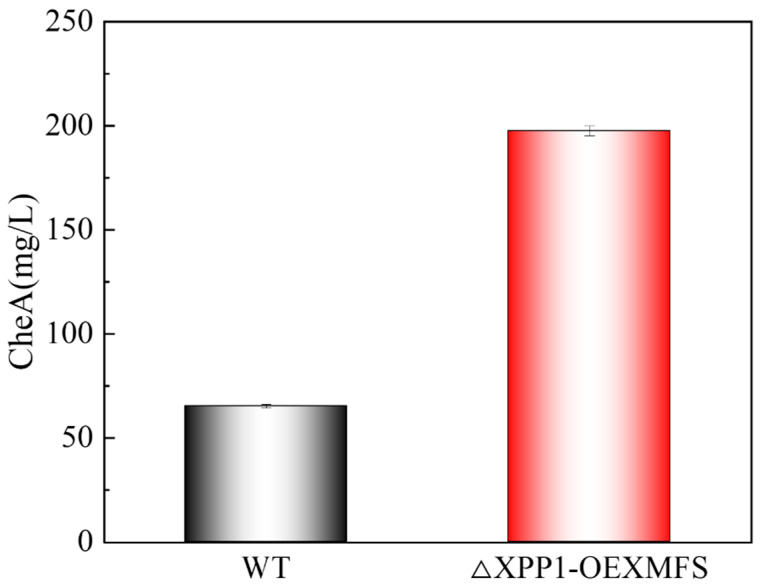
Scale-up experiment for the production of CheA using potato waste.

Finally, we used a 5 L airlift fermenter (with a volume of 7 L) to carry out a fermentation experiment with potato waste, in which an aeration rate of 0.2 vvm replaced the rotational speed of 180 rpm under optimal conditions. The air rise culture mode makes it easier to achieve the mixing of the substrate and strain, reduces the damage to cells caused by shear stress, and improves the oxygen delivery efficiency at the same time. Under optimized conditions, we set the ventilation rate to 0.2 vvm instead of 180 rpm to better simulate an industrial production environment [43,44]. The CheA yield by the engineered strain was 197.58 mg/L at 10 d compared to 65.35 mg/L for the WT strain (Figure 8). Using potato starch industrial waste residue as the fermentation substrate not only reduces industrial waste residue emissions and reduces environmental pollution, but also improves the utilization efficiency of resources [45,46,47]. This provides a new idea and method for the resource utilization of agricultural waste. It was further shown that the engineered strain had a stable performance, and the production cost was reduced while the CheA yield was increased. This study provided more substantial theoretical data for the subsequent large-scale and resource-based production of CheA.

### 3.10. Quantitative Analysis of Key Genes After Optimization

**Figure 9:**
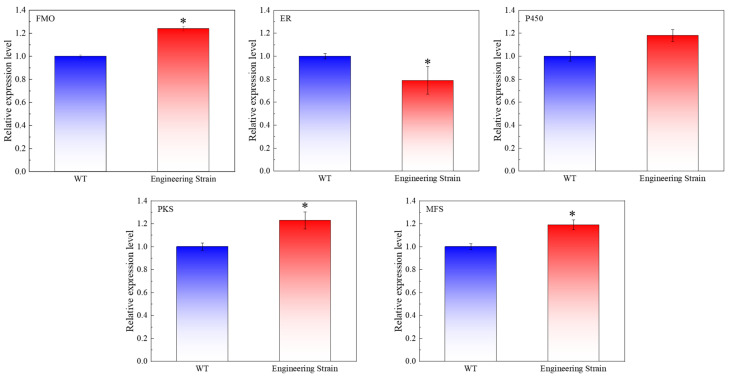
Quantitative analysis of key genes (MFS, PKS, P450, FMO and ER) after optimization. (*: *p* < 0.05).

To explore the effect of fermentation condition optimization on CheA biosynthesis by the engineered strains, we selected a fermentation time of 5 d to determine the expression levels of key CheA synthesis genes (*CgP450*, *CgFMO*, *CgPKS*, and *CgER*) and transport genes (*CgMfs*) (Figure 9). A comparative analysis revealed that, under optimized fermentation conditions, the expression levels of *CgFMO*, *CgPKS*, and *CgMfs* in the engineered strain were significantly higher than those in the wild-type (WT) strain. This upregulation suggests that the optimized conditions enhanced the transcriptional activation of these genes, potentially by modulating the activity of regulatory proteins or stabilizing mRNA transcripts [47,48,49]. In contrast, the expression of *CgER* was markedly downregulated in the engineered strain under optimized conditions, indicating that the reduction step in CheA biosynthesis may be less critical or even rate-limiting under these conditions. Additionally, the expression of *CgP450* remained unchanged, implying that the oxidation reactions catalyzed by this enzyme were not significantly affected by the fermentation optimization.

## 4. Conclusions

In the present work, to increase CheA yield, a genetically modified transformant with *CgXpp1* knockout and *CgMfs* overexpression was constructed, which significantly increased the CheA yield from 63.19 to 265.93 mg/L under fermentation in PDA medium. Also, in order to reduce production costs, the engineered strain was selected to use potato starch industrial waste as its sole substrate, and the addition of Cu^2+^ boosted CheA production to 176.92 mg/L. The response surface method was used to optimize the liquid fermentation conditions as follows: a temperature of 28 °C, initial pH of 6.0, rotation speed of 180 rpm, and the addition of 50 g/L dry matter potato waste. Furthermore, under the optimal conditions, the engineered *CgXpp1* knockout and *CgMfs* overexpression strain could stably produce CheA with a yield of 197.58 mg/L, which was 3.12-fold higher than that of the WT strain. This study provided a theoretical basis for the industrialization of CheA production. With further research and technological development, greater breakthroughs are expected, opening up new roads for environmental protection and biotechnological applications.

## Figures and Tables

**Table 1:** Determination of nutrient content of potato starch industrial waste (g/L).

	Discarded Potatoes	Starch Processing Wastewater
Crude fibre	27.25 ± 0.18	9.74 ± 0.56
Protein	5.73 ± 0.13	17.2 ± 0.23
Fat	0.62 ± 0.34	0.35 ± 0.10
Ash	1.85 ± 0.16	1.25 ± 0.25
Carbohydrates	26.8 ± 2.34	11.3 ± 0.47

**Table 2:** Experimental design scheme and results of response surface.

Run	Temperature (°C)	pH	Speed (rpm)	Amount of Substrate (g/L)	CheA (mg/L)
1	0	0	0	0	193.84
2	1	−1	0	0	147.01
3	−1	1	0	0	146.28
4	0	1	−1	0	141.78
5	1	0	0	1	144.07
6	−1	0	1	0	145.09
7	0	−1	0	1	142.81
8	0	0	0	0	194.27
9	−1	−1	0	0	146.65
10	0	−1	−1	0	143.46
11	1	0	−1	0	140.47
12	0	1	1	0	143.11
13	1	0	0	−1	143.89
14	0	1	0	−1	142.63
15	0	0	1	1	142.86
16	0	0	0	0	193.07
17	0	0	−1	−1	142.81
18	0	0	−1	1	142.27
19	1	1	0	0	142.14
20	0	0	1	−1	146.41
21	0	−1	0	−1	145.75
22	0	1	0	1	141.07
23	0	0	0	0	193.31
24	−1	0	0	1	143.28
25	1	0	1	0	148.63
26	−1	0	0	−1	148.87
27	0	0	0	0	194.15
28	0	−1	1	0	146.59
29	−1	0	−1	0	148.99

## Data Availability

The original contributions presented in this study are included in the article/Supplementary Materials. Further inquiries can be directed to the corresponding authors.

## Supplementary Materials

The following supporting information can be downloaded at: https://www.mdpi.com/article/10.3390/foods14050842/s1, Table S1. Primers designed for *C. globosum* W7 in this work; Table S2. Regression and variance analysis table.
