# Supplementary material for: Construction of an Efficient Engineered Strain for Chaetoglobosin A Bioresource Production from Potato Starch Industrial Waste
Source: Foods. 2025 Feb 28;14(5):842. doi: 10.3390/foods14050842 (PMC11898925; doi:10.3390/foods14050842)
Supplement: Supplementary file 1 [file foods-14-00842-s001.zip › foods-3433973-supplementary.pdf]

Construction of an Efficient Engineered Strain for Chaetoglobosin A Bioresource Production from Potato Starch Industrial Waste

Kai Zhang [1,2,3,†], Shanshan Zhao [1,3,4,*,†], Zhengran Wang [4], Ming Cheng [3], Wan Wang [3] and Qian Yang [3,*]

[1] Donghai Laboratory, Zhoushan 316021, China
[2] School of Life Science, Ludong University, 186 Hongqi Road, Yantai 264025, China
[3] School of Life Science and Technology, Harbin Institute of Technology, Harbin 150080, China
[4] Institute of Marine Biology and Pharmacology, Ocean College, Zhejiang University, Zhoushan 316021, China
* Correspondence: zhaoshanshan5612@163.com (S.Z.); yangq@hit.edu.cn (Q.Y.)
† These authors contributed equally to this work.

Table S1. Primers designed for *C. globosum* W7 in this work.

Primer	Sequence
mcheOF-*CgMfs*	5'-CCGTCGACCTCGACTCTAGAGGATCATGGAGGAAAAGAAAGAA GAAAACAA-3'
mcheOR-*CgMfs*	5'-TCGCCCTTGCTCACCATATCGAATTCTCATGCCTCCACGTCTAGG CCA-3'
HPF	5'-TGCTGAGGTCCCTCAGTCCCTG-3'
HPR	5'-CGTCCTGCAGGGAGGAGTCCT-3'
actin-F	5'-TCATCGACAATGGCTCCGGTATG-3'
actin-R	5'-GCTCGTTGTAGAAGGTGTGATGC-3'
CgMfs-QF1	5'-TTTTTTACCGTCCTTAGCGTCTTG-3'
CgMfs-QR1	5'-TTCCCGCGCCTTTTCCA-3'
RT-ER-F	5'-GTCGGGAGTTTGGGCATTCA-3'
RT-ER-R	5'-ACACCTCGTCTGCGCCATAA-3'
RT-FMO-F	5'-ACTTCGCCGCCGAATCATCC-3'
RT-FMO-R	5'-AGCAGAATGGGCAGGGCAAA-3'
RT-PKS-F	5'-CCGATTGACGGCAAAGGC-3'
RT-PKS-R	5'-GCAGCAATGGCGAGAGGAA-3'
RT-P450-F	5'-CGGGGGTGCTTTTTTACAT-3'
RT-P450-R	5'-GGGTTGACTCGGGCTGACT-3'

Table S2 Regression and variance analysis table

Source	Sum of squares	df	Mean Square	F value	*p*-value	significance
Model	10190.03	14	727.86	6144.16	< 0.0001	significant
A-pH	14.04	1	14.04	118.51	< 0.0001	
B-Temperature	19.40	1	19.40	163.79	< 0.0001	
C-Speed	13.90	1	13.90	117.30	< 0.0001	
D-substrate	16.32	1	16.32	137.75	< 0.0001	
AB	5.06	1	5.06	42.72	< 0.0001	
AC	36.37	1	36.37	307.04	< 0.0001	
AD	8.33	1	8.33	70.31	< 0.0001	
BC	0.81	1	0.81	6.82	0.0205	
BD	0.48	1	0.48	4.04	0.0641	
CD	2.26	1	2.26	19.05	0.0006	
A^2	3475.56	1	3475.56	29338.62	< 0.0001	
B^2	4106.27	1	4106.27	34662.72	< 0.0001	
C^2	3977.17	1	3977.17	33572.94	< 0.0001	
D^2	4211.71	1	4211.71	35552.75	< 0.0001	
Residual	1.66	14	0.12			
Lack of Fit	0.56	10	0.056	0.21	0.9803	not significant
Pure Error	1.09	4	0.27			
Cor Total	10191.69	28				
